# Investigating the relationship between particulate matter and inflammatory biomarkers of exhaled breath condensate and blood in healthy young adults

**DOI:** 10.1038/s41598-021-92333-6

**Published:** 2021-06-21

**Authors:** Morteza Seifi, Noushin Rastkari, Mohammad Sadegh Hassanvand, Kazem Naddafi, Ramin Nabizadeh, Shahrokh Nazmara, Homa Kashani, Ahad Zare, Zahra Pourpak, Seyed Yaser Hashemi, Masud Yunesian

**Affiliations:** 1 Department of Environmental Health Engineering, School of Public Health, Tehran University of Medical Sciences, Tehran, Iran; 2 Center for Air Pollution Research (CAPR), Institute for Environmental Research (IER), Tehran University of Medical Sciences, Tehran, Iran; 3 Department of Research Methodology and Data Analysis, Institute for Environmental Research, Tehran University of Medical Sciences, Tehran, Iran; 4 Immunology, Asthma and Allergy Research Institute, Tehran University of Medical Sciences, Tehran, Iran

**Keywords:** Immunology, Environmental sciences, Environmental social sciences, Biomarkers

## Abstract

Inflammatory biomarkers in exhaled breath condensate (EBC) are measured to estimate the effects of air pollution on humans. The present study was conducted to investigate the relationship between particulate matter and inflammatory biomarkers in blood plasma and exhaled air in young adults. The obtained results were compared in two periods; i.e., winter and summer. GRIMM Dust Monitors were used to measure PM_10_, PM_2.5_, and PM_1_ in indoor and outdoor air. A total of 40 healthy young adults exhaling air condensate were collected. Then, biomarkers of interleukin-6 (IL-6), Nitrosothiols (RS-NOs), and Tumor necrosis factor-soluble receptor-II (sTNFRII) were measured by 96 wells method ELISA and commercial kits (HS600B R&D Kit and ALX-850–037-KI01) in EBC while interleukin-6 (IL-6), sTNFRII and White Blood Cell (WBC) were measured in blood plasma in two periods of February 2013 (winter) and May 2013 (summer). Significant association was found between particulate matter and the white blood cell count (p < 0.001), as well as plasma sTNFRII levels (p-value = 0.001). No significant relationship was found between particulate matter with RS-NOs (p = 0.128), EBC RSNOs (p-value = 0.128), and plasma IL-6 (p-value = 0.167). In addition, there was no significant relationship between interleukin-6 of exhaled air with interleukin-6 of plasma (p-value < 0.792 in the first period and < 0.890 in the second period). sTNFRII was not detected in EBC. Considering the direct effect between increasing some biomarkers in blood and EBC and particulate matter, it is concluded that air pollution causes this increasing.

## Introduction

Many epidemiologic studies have been conducted on the health effects of air pollution. The outcomes have shown that exposure to air pollution is associated with a range of acute and chronic health effects, from minor physiological disorders to death from respiratory and cardiovascular diseases^1–5^. Some results emphasize that long-term exposure to particulate matter can reduce the lifetime of a person^6,7^. Increasing the concentration of PM_10_ increases the risk of respiratory death in children, affects lung function and exacerbates asthma, and causes other respiratory symptoms such as coughing and bronchitis in children^8–11^. PM_2.5_ has a serious impact on health, increasing the risk of death from respiratory and cardiovascular diseases and lung cancer^12,13^. Toxicological studies have shown that fine particles are more related to respiratory and cardiovascular diseases than larger particles^14–19^. The evaluation of biomarkers in the blood is one of the methods for estimating the effects of air pollution on humans. Another new method to collect exhaled breath condensates in estimating the effects of air pollution^20^. Exhaled Breath Condensate (EBC) is a non-invasive, appropriate, and inexpensive method used for scientific research and diagnosis of respiratory diseases. the formation of EBC after condensing droplets released by airflow from the airway lining fluid and being diluted by alveolar air and mixed by volatile molecules of the airway tract. It is also possible to detect some effects through collecting exhaled air condensate and measurement of biomarker changes^14,20–26^. EBC identifies the fluid composition of the respiratory tract and helps to identify and to diagnose the diseases. The primary components of EBC include collected aerosols in the respiratory tract, distilled vapors around aerosols, and evaporated gas dissolved in distilled water vapor in the respiratory tract^6,27–30^. EBC, which is mainly composed of molecules in the respiratory tract diluted with water vapors, contains simple ions such as hydrogen ions, hydrogen peroxide, proteins, cytokines, eicosanoids, and macromolecules such as mucin, phospholipid, and DNA. EBC is used to analyze exhalation air; e.g., estimating blood glucose^21,22,31^. Studies have shown that the concentration of markers in EBC is higher than normal in some diseases. Detection of these compounds depends on the available technology for analysis^23,32–37^. The studies to examine the biomarkers of exhaled condensates have been remarkably advanced. Hence, in various studies, new macromolecules have been identified in exhaled air.

Patel et al. investigated the emissions from traffic and exhaled breath markers in teenagers in New York City^38^. In the study, the air pollutants including nitrogen dioxide, ozone, and particulate matter were measured and an association was obtained between these ambient air pollutants and exhaled breath biomarkers. It has been shown that in all participants, a 1–5 day increase in exposure to black carbon can reduce the pH of the exhaled breath condensates (EBC), which causes inflammation of the respiratory tract and increases the 8-isoprostane, which ultimately increases the oxidative stress. An increase of 1–5 days in exposure to nitrogen dioxide also increases the 8-isoprostane. Ozone and fine particles are also unevenly related to exhaled breath biomarkers. This study showed that there is no difference between asthmatic and non-asthmatic individuals in exposure to air pollutants, and short-term exposure to traffic-pollutants may increase inflammation and oxidative stress in respiratory pathways^38^.

In a study conducted by Manney et al. (2015) in the UK on the association between exhaled breath condensate and nitrate + nitrite levels with ambient coarse particle exposure in subjects with airways disease, was found an association between EBC NOx as a marker of oxidative stress and exposure to ambient coarse particles at central sites. The lack of association between PM measures is more indicative of personal exposures (particularly indoor exposure)^39^.

Huang et al. in 2012 investigated changes in air pollution levels during the Beijing Olympics related to biomarkers of inflammation and thrombosis in healthy young adults and resulted that from the pre-Olympic to the during-Olympic period, concentrations of particulate and gaseous pollutants decreased substantially (-13% to -60%). The changes were associated with measures of cardiovascular physiology and acute changes in biomarkers of thrombosis and inflammation in healthy young persons^40^.

Delfino et al. studied a susceptible population for the effect of exposures of air pollution and circulating biomarkers in 2009. They found that air pollutants caused by traffic were associated with increases in platelet activation, increasing in systemic inflammation, and decreasing in erythrocyte antioxidant enzyme activity, which may be partly behind increases in systemic inflammation caused by air pollution. Differences in association by organic carbon fraction, seasonal period, and particle size suggest the importance of components carried by ultrafine particles^41^. The mentioned studies helped us to be able to select healthy people to study the purpose of this study, and also these studies helped us to select the type of biomarkers.

Little is known about the association between exposure to particulate matter and EBC in highly polluted megacity such as Tehran. The purpose of the current study is to investigate the relationship between particulate matter and inflammatory biomarkers of exhaled breath condensate and blood in healthy young adults.

## Materials and methods

### Study design and participants

This study was conducted in two 6-day periods of February 2013 (winter) and May 2013 (summer). A total of 40 healthy young adults were recruited initially. However, the study ended with 36, with the withdrawal of four young men. Each of the volunteers before entering the study stated their consent by signing a consent form. Volunteers could freely leave the study at any phase. All experimental protocols and the study were approved by the ethics committee of the institute for environmental research (IER) of Tehran University of Medical Sciences. All methods were carried out per relevant guidelines and regulations. We appreciate the sincere cooperation of the administrations of Hejrati School, where the sampling phase of the study was conducted. Informed consent was obtained from participants. Our focus has not been on the disease but only on biomarkers in exhaled air and blood and contact with suspended particles.

### Study inclusion and exclusion criteria

Inclusion criteria for healthy young adults were the age of 16 years old, lack of illness, permanent residence in a centralized location, voluntary participation, and not smoking. Exclusion criteria for healthy young adults were a failure to participate during the study, infection in one week before blood sampling, leaving the boarding school in 6 days leading to sampling and death.

### Schedule of the desired monitoring

As mentioned before, the main objective of the study is to investigate the association between exposure to particulate matter and biomarkers. For this purpose, the outdoor and indoor particulate matter monitoring program was designed and implemented. So, the program of indoor and outdoor particulate matter monitoring was designed to determine the relationship between the lags of exposure to markers. The purpose of indoor and outdoor particulate matter monitoring was to determine the relationship between exposures to particulate matter at 0–144 h (0 to 6 days exposure) in healthy young adults. For each 6-day period, the PM concentration of indoor and outdoor was measured continuously by direct reading equipment and the device recorded the data every hour in two 6-day periods. Finally, for each 6-day period, 144 hourly samples were taken by a direct reading equipment for any size of airborne particles.

#### Indoor and outdoor particulate matter mass concentration monitoring

Data on outdoor and inside the school dormitory particulate matter exposure were measured in two 6-day periods of February 2013 (winter) and May 2013 (summer). Exposure to air pollutants is due to exposure to indoor and outdoor air pollutants. Since people usually spend more than 80% of their time in the interior environment^23^, in addition to measuring outdoor pollutants, indoor air should be monitored for estimating the actual exposure. In this study, the indoor and outdoor mass concentrations of PM_10_, PM_2.5,_ and PM_1_ were measured simultaneously using a a piece of direct-reading equipment (GRIMM dust monitors: model 107/1 for outdoor air monitoring and model 108/1 for indoor air monitoring) according to Eq. 1^42^.

1 $$\mathrm{Y}=\frac{\left(\mathrm{C}1\mathrm{*}\mathrm{t}1\right)+\left(\mathrm{C}2\mathrm{*}\mathrm{t}2\right)+\dots +(\mathrm{C}\mathrm{n}\mathrm{*}\mathrm{t}\mathrm{n})}{\mathrm{t}1+\mathrm{t}2+\dots +\mathrm{t}\mathrm{n}}$$

#### Estimation of adult’s exposure

To estimate the exposure of young adults to PM_10_, PM_2.5_, and PM_1_, at the beginning of each 6 days, a notepad was given to all people and they were asked to register their hourly or even every 10-min attendance at different parts of the school. At the end of each working day, the attendance times were collected. As a result, the exposure level for each person at any time was equivalent to the concentration of particulate matter in the environment (indoor and outdoor) the person was at the corresponding time.

#### Demographic and clinical characteristics of participants

The demographic and clinical characteristics of participants such as age, smoking status, history of diseases, and drug use were collected using a questionnaire and by personal interviewing by a physician. Clinical characteristics of participants (such as sex (male), age, smoking, cardiovascular history (Coronary artery bypass graft or angioplasty, positive angiogram or stress test, hypertension, hypercholesterolemia, current angina pectoris, pacemaker or defibrillator, cardiac arrhythmia), other medical history (type II diabetes, COPD, transient ischemic attack, chronic bronchitis, hay fever), medications (ACE inhibitors, HMG CoA reductase inhibitors (statins), platelet aggregation inhibitors, aspirin, calcium channel blockers, clopidogrel bisulfate (Plavix), antihyperlipidemic medication, antihyperlipidemic medication, anti-arrhythmics and anti-arrhythmics) were examined by a general practitioner 30 min before each blood sampling in site. Of those, who had infectious diseases within a week leading up to the sampling, blood samples, and EBC was not taken^43^.

#### Collection and processing of exhaled breath condensates

**Figure 1:**
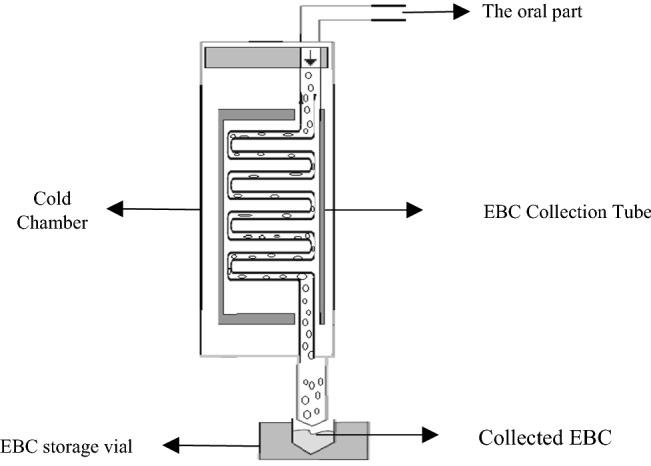
schematic of the EBC collector designed in this study.

Sampling was done while people were sitting on the chair. In this situation, the inhalation was through the nose and exhalation was through the mouth, and exhalation entered the collector through the mouth. EBC was collected continuously after 10–15 min in a device by the rate of 1–3 ml as a mixture of solid and liquid. The device was designed to collect EBC, which was stored at − 80℃ in propylene vials after collection. Samples were taken in two periods to determine the level of biomarkers and exhaled breath condensates. For this purpose, 6 days before the sampling, the indoor and outdoor particulate matters were measured and at the end of day 6, samples were taken from adults. Since the infection can lead to an increase in inflammatory mediators, the history of infection in the week before the sampling was evaluated by a physician and people with the infection were excluded from the study. Samples were collected and stored in a cold box at a temperature of − 20 ℃ after collection and immediately transferred to the laboratory. Next, they were subjected to several aliquots and stored at − 80 ℃ in the laboratory freezer after encoding. Figure 1 presents a schematic of the EBC collector designed in this study^44^.

#### Measurement of blood biomarkers

The measurement of blood biomarkers is described in detail elsewhere^43^. WBC was performed on freshly collected blood samples. Plasma aliquots were immediately stored at −70 ℃ until being tested and blood samples were centrifuged for 15 min at 4 ℃. Three biomarkers were considered in this study: Interleukin-6 (IL-6), white blood cells (WBC), and tumor necrosis factor-soluble receptor-II (sTNF-RII). IL-6 and sTNF-RII were analyzed with enzyme-linked immunosorbent assay (Quantikine, R&D Systems) at Immunology, Asthma, and Allergy Research Institute, Tehran University of Medical Sciences (Tehran, Iran). WBC of whole blood was counted using an automatic hematological analyzer (CellDyn 4000, Abbott). All samples were analyzed in duplicate to ensure reproducibility. The blood sampling tube was made of plastic. Centrifuges are used for biomarkers (The g force of the centrifuge was 1100). No centrifuge was used for white blood cells. In this study, both EDTA and citrate tubes were used. EDTA tubes were used to measure white blood cells and citrate tubes were used to measure blood biomarkers.

#### Exhaled biomarkers measuring

The concentrations of IL-6 and RS-NOs were measured by method ELISA 96 wells (Enzyme-linked immunosorbent assay) and using commercial kits^29^.

#### RS-Nos measurements

RS-NOs were measured using a commercially available colorimetric assay kit (Oxonon, Emeryville, CA). The assay is based upon the classic reaction of Saville and Griess^40,41^. In essence, a cleavage reaction breaks the S–N bond of RS-NOs releasing NO, which oxides rapidly to NO2. NO2 is then detected calorimetrically using the Griess reaction. Briefly, 200 ml of EBC were used for each assay, and 50 µl of Griess 1 was added followed by 50 µL of Griess 2. The product was measured spectrophotometrically (Model AR 8003; Labtech Int. Ltd., Uckfield, UK) at 540 nm. A standard curve of nitrosogluthatione (GS-NO) was performed for each assay. The detection limit of the kit is 0.025 µM. Levels of RS-NOs were determined by interpolation from the known standard curve and were expressed as µM concentration. All the samples were run in duplicate, and mean values were used for subsequent analysis^35,45^.

### Statistical analysis

The concentration of PM_10_, PM_2.5_, and PM_1_ and the minimum, maximum, mean, standard deviation (SD), and quartiles for IL-6 EBC, RS-Nos EBC, IL-6 plasma, and sTNFRII plasma were reported in both sampling periods. To assess the correlation between the IL-6 EBC and IL-6 plasma, Spearman correlation coefficient was used. Paired-samples t-test and Wilcoxon signed-rank test were applied to assess the changes between the levels of biomarkers in the two sampling periods. Analyses were performed using IBM SPSS Statistics for Windows (IBM Corp. Released 2011, Version 20.0. Armonk, NY: IBM Corp) and p-value < 0.05 was considered statistically significant. In this study, Bonferroni correction is a method used to adjust the problem of multiple comparisons.

## Results

Temperature, Relative humidity, and wind speed are shown in Table1. The average concentrations of indoor and outdoor particulate matter during the sampling periods are shown in Table 2. The results of IL-6 measurement are presented in Tables 3 and 4. The average concentration of exposure to particulate matter measured in a school dormitory in the first sampling period (winter) is presented in Table 3 and the second sampling period (summer) are presented in Table 4. According to Table 3, the concentration of IL-6 is between 0.3 and 2.3 and the average is 1.08 pg/ml. Percentiles of 0.25, 0.5, and 0.75 were 0.76, 0.96, and 1.33, respectively. The EBC volume was measured between 1.2 and 3.5 ml and an average of 2.3 ml per person. The findings also showed that the ratio of positive to negative samples and missing samples to total samples was 2.36 and 0.39, respectively.

**Table 1:** Temperature, relative humidity and wind speed.

Variable	Temperature (°C)	Wind velocity (m/s)	Relative humidity (%)
**Outdoor**			
Min	− 0.3	0.13	43
Max	37	9.2	51
Average	22.5	2.9	38
**Indoor**			
Min	24	–	21
Max	28	–	38.5
Average	26.51	–	29.96

**Table 2:** The average concentration of indoor and outdoor particulate matter during the sampling periods.

Variable	Outdoor (µg/m^3^)	Indoor (µg/m^3^)
**First sampling**		
PM_10_	61.4 ± 9.25	46.68 ± 6.25
PM_2.5_	24.34 ± 5.12	15.78 ± 2.34
PM_1_	16.56 ± 3.68	8.01 ± 1.14
**Second sampling**		
PM_10_	66.83 ± 10.74	93.5 ± 11.56
PM_2.5_	21.65 ± 4.61	18.42 ± 2.33
PM_1_	15.15 ± 2.96	6.09 ± 1.02

**Table 3:** The average concentration of exposure to particulate matter measured in a school dormitory in the first sampling period (March-winter).

Variable	First day	Second day	Third day	Fourth day	Fifth day	Sixth day	An average of 2 days	An average of 3 days	An average of 4 days	An average of 5 days	An average of 6 days
**PM (µg/m**^**3**^**)**											
PM_10_	43.32 ± 5.22	56.7 ± 6.81	43.67 ± 4.67	43.21 ± 4.56	49.14 ± 5.68	62.63 ± 6.12	50.1 ± 5.42	47.9 ± 4.92	46.73 ± 3.98	47.21 ± 5.25	48.72 ± 4.57
PM_2.5_	16.41 ± 1.85	17.45 ± 2.21	16.48 ± 1.85	13.38 ± 2.12	17.79 ± 3.25	22.62 ± 3.78	16.93 ± 2.95	16.78 ± 2.23	15.93 ± 3.24	16.3 ± 3.37	16.92 ± 4.12
PM_1_	9.48 ± 1.17	8.57 ± 0.97	9.73 ± 1.23	6.97 ± 0.5	9.85 ± 1.31	11.92 ± 1.87	9.02 ± 1.03	9.26 ± 2.12	8.69 ± .89	8.92 ± .56	9.21 ± 1.46

**Table 4:** The average concentration of exposure to particulate matter measured in a school dormitory in the second sampling period(June-summer).

Variable	First day	Second day	Third day	Fourth day	Fifth day	Sixth day	An average of 2 days	An average of 3 days	An average of 4 days	An average of 5 days	An average of 6 days
**PM (µg/m**^**3**^**)**											
PM_10_	106.2 ± 14.56	94.58 ± 12.24	87.68 ± 9.87	61.27 ± 8.95	61.27 ± 10.68	91.65 ± 12.21	100.6 ± 14.52	96.29 ± 8.72	78.45 ± 6.53	78.45 ± 7.65	78.45 ± 6.58
PM_2.5_	27.13 ± 5.29	24.06 ± 4.78	14.82 ± 6.73	9.9 ± 1.45	17.28 ± 2.26	21.37 ± 3.65	25.6 ± 3.44	22 ± 4.01	18.98 ± 4.17	18.64 ± 3.98	19.09 ± 3.21
PM_1_	9.43 ± 2.58	8.56 ± 2.23	5.95 ± 0.40	4.44 ± 0.68	5.74 ± 0.97	8.74 ± 1.32	9 ± 1.01	7.98 ± 1.05	7.09 ± 1.2	6.82 ± 1.01	7.14 ± .97

Description of measured biomarkers in the first sampling period (winter) and the second sampling period (summer) is presented in Tables 5 and 6, respectively.

**Table 5:** Description of measured biomarkers in the first sampling period (March-winter).

Variable	Samples	Average	Median	SD	Max	Min	CV	Quartile		
								Q_1_	Q_2_	Q_3_
**Biomarker**										
IL-6 EBC (pg/ml)	36	0.56	0.55	0.22	1.17	0.05	0.39	0.41	0.54	0.7
RS-Nos EBC (µM)	36	0.97	0.69	0.89	3.53	0	0.91	0.31	0.7	1.55
IL-6 plasma (pg/ml)	36	7.2	5.5	6.98	3.53	0.36	0.97	1.7	6	1.25
sTNFRII plasma (pg/ml)	36	1730	1705	569	3083	652	0.32	1257	1735	2060
WBC (k/µl)	36	6.2	2.9	1.3	9.7	4.4	0.2	5.04	6.15	7.20

**Table 6:** Description of measured biomarkers in the second sampling period (June-summer).

Variable	samples	Average	Median	SD	Max	Min	CV	Quartile		
								Q_1_	Q_2_	Q_3_
**Biomarker**										
IL-6 EBC (pg/ml)	36	1.08	0.96	0.47	2.3	0.3	0.43	0.73	0.96	6.5
RS-Nos EBC (µM)	36	1.13	1.02	0.93	4.47	0	0.82	0.37	1.02	1.5
IL-6 plasma (pg/ml)	36	13.36	1.64	41.7	241	0.13	3.12	0.67	2	5
sTNFRII plasma (pg/ml)	36	1914	1827	644	3492	0.13	0.34	0.67	1871	2376
WBC (k/µl)	36	6.7	3.5	1.45	3492	4.8	0.21	5.5	6.5	7.8

### Investigating the relationship between exhaled breath biomarkers and particulate matter

**Figure 2:**
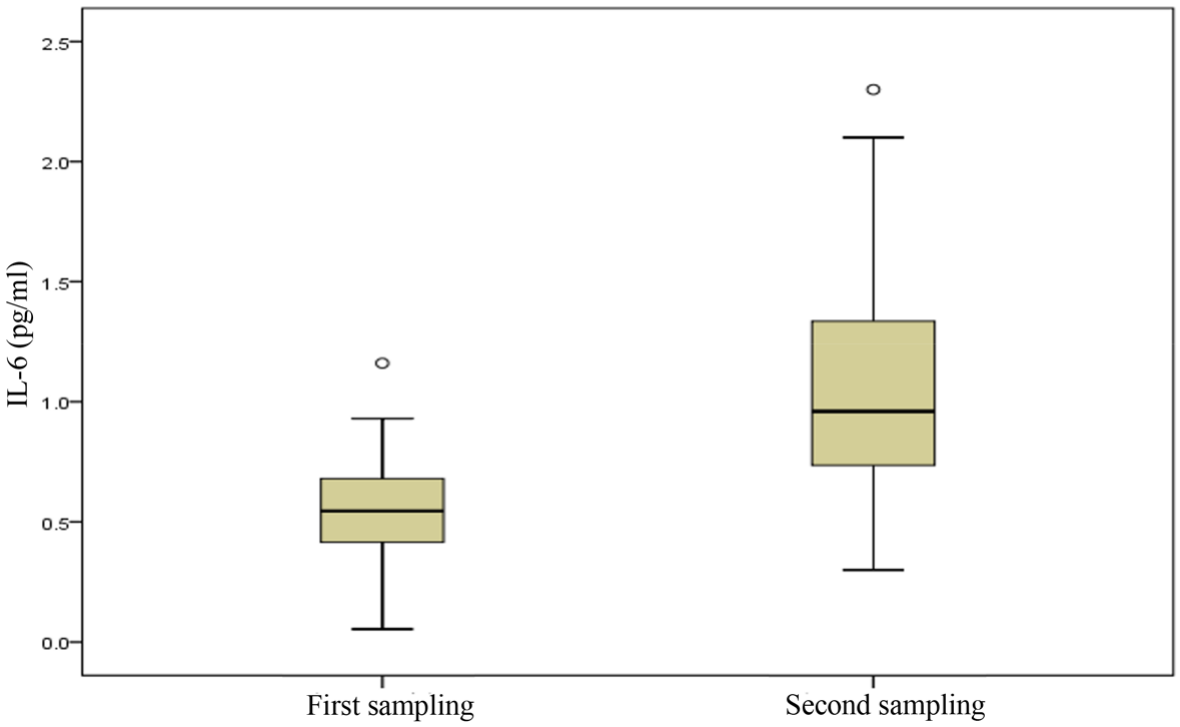
The concentration of IL-6 in EBC in two sampling periods.

**Figure 3:**
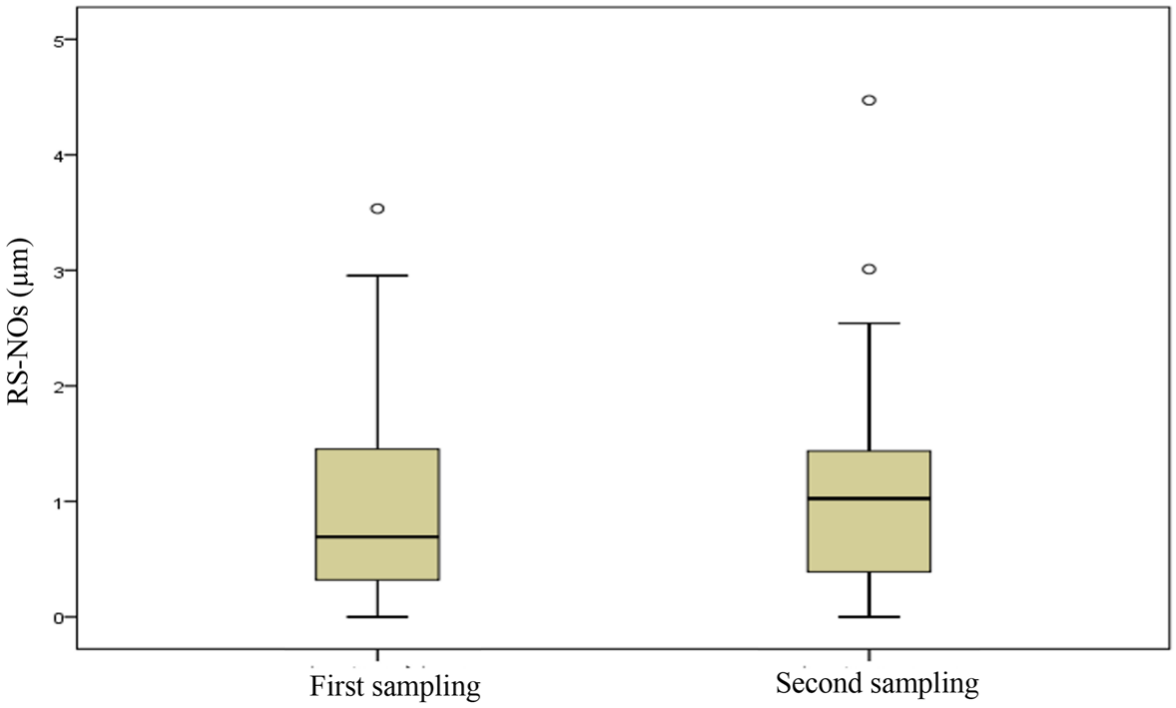
The concentration of RS-NOs in EBC in two sampling periods.

There was a significant rise in exhaled IL-6 in the second sampling period (p-value < 0.005 Bonferroni-correction. In comparison, there was no significant difference in exhaled RS-NOs concentration in two sampling periods (p-value = 0.64 Bonferroni-correction). The concentration of IL-6 in EBC in two sampling periods is presented in Fig. 2 and the concentration of RS-NOs in EBC in two sampling periods is presented in Fig. 3. In addition, sTNFRII was not detected in EBC. As can be seen in Tables 3 and 4, the concentration of PM_10_ in the two periods differs by eighty percent and shows the effect.

### Investigating the relationship between blood biomarkers and particulate matter

**Figure 4:**
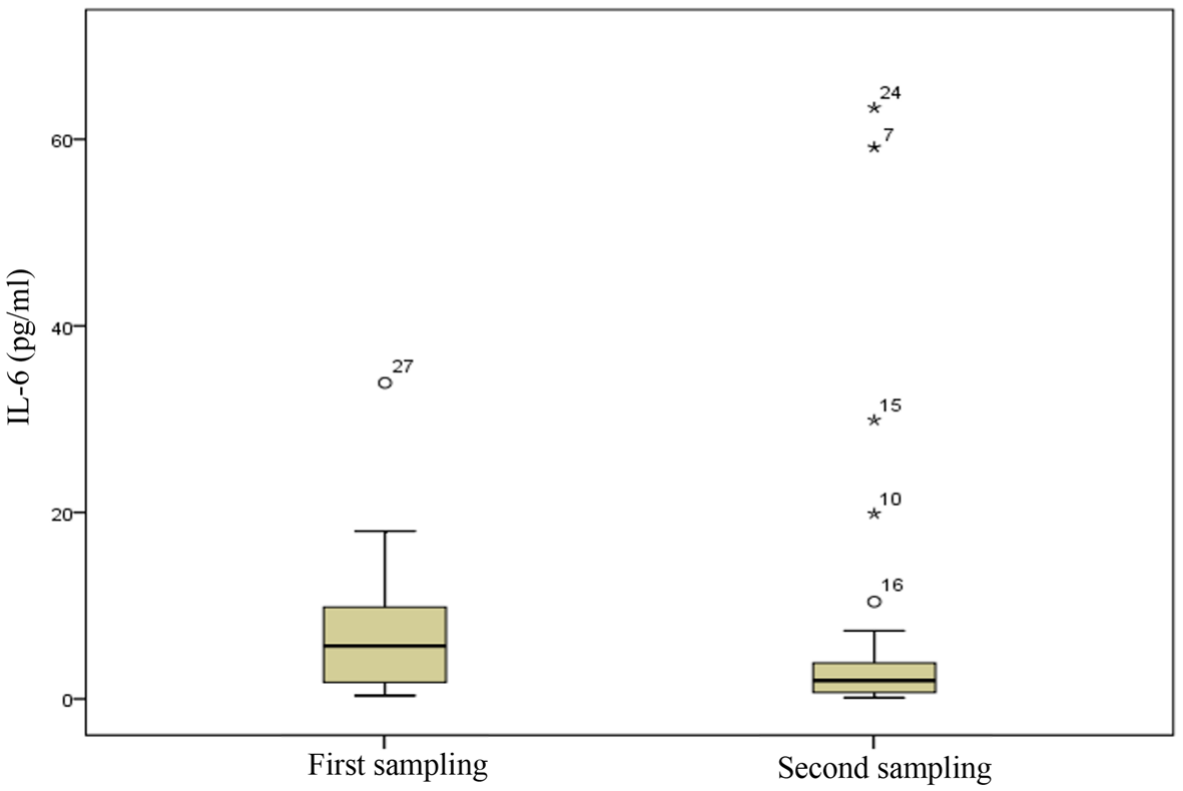
The concentration of IL-6 in blood in two sampling periods.

**Figure 5:**
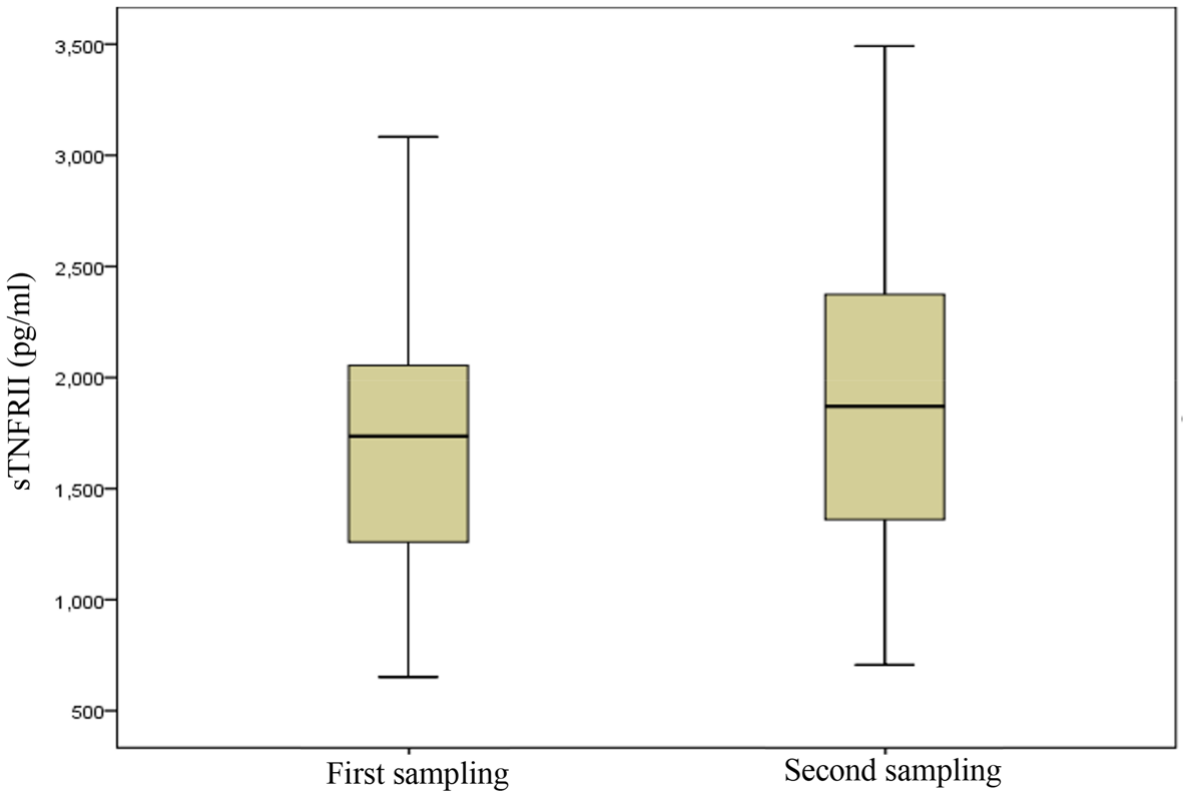
The concentration of sTNFRII in blood in two sampling periods.

**Figure 6:**
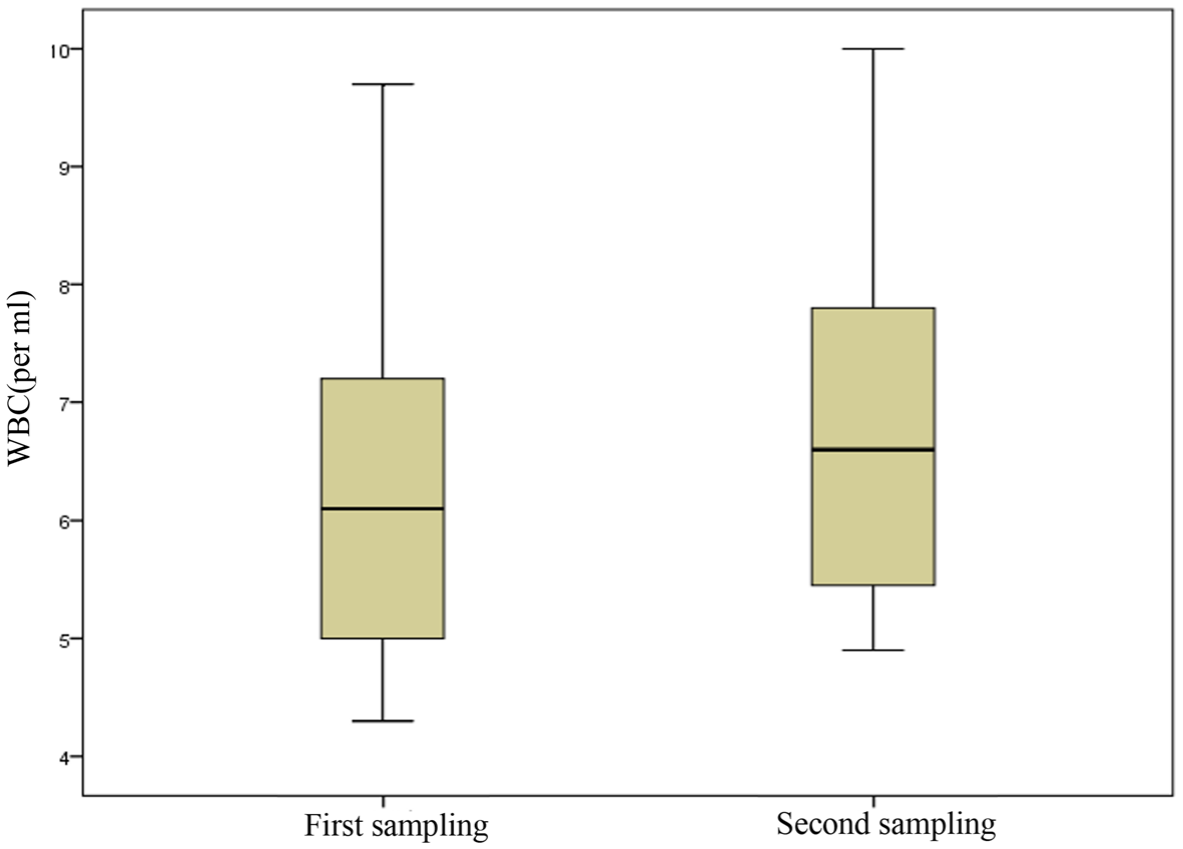
The concentration of WBC in blood in two sampling periods.

No significant relationship was found in blood IL-6 in two periods (p-value = 0.835 Bonferroni-correction) The measurement of IL-6 in blood in two sampling periods in Fig. 4. There were significant sTNFRII and WBC of blood in the second sampling period (p-value < 0.005 Bonferroni-correction). The measurement of sTNFRII and WBC in blood in two sampling periods in Fig. 5 and Fig. 6. Power analysis was performed using software PS Power and Sample Size version 3.0. The minimum value was 0.8 for RS-Nos and other parameters was more than 0.81.

### Investigating the relationship between exhaled breath biomarkers and blood biomarkers

**Figure 7:**
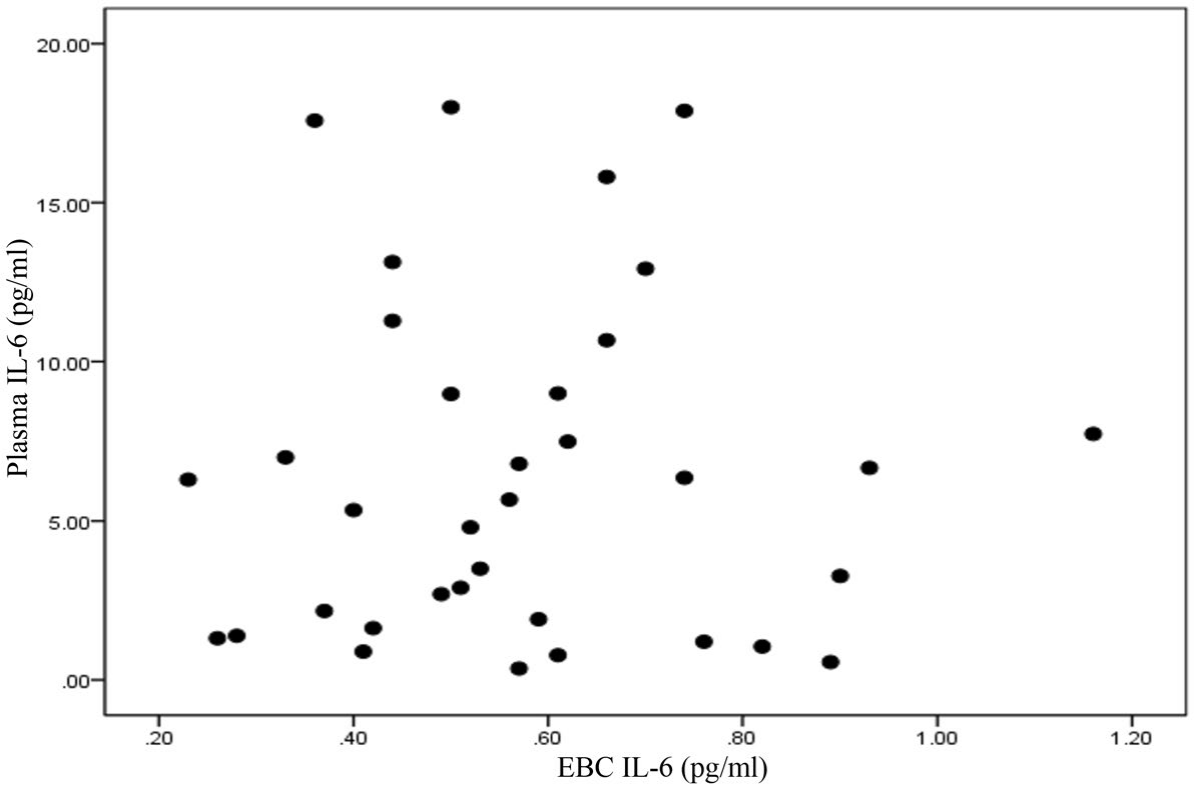
The concentration of IL-6 in EBC and plasma first sampling periods.

**Figure 8:**
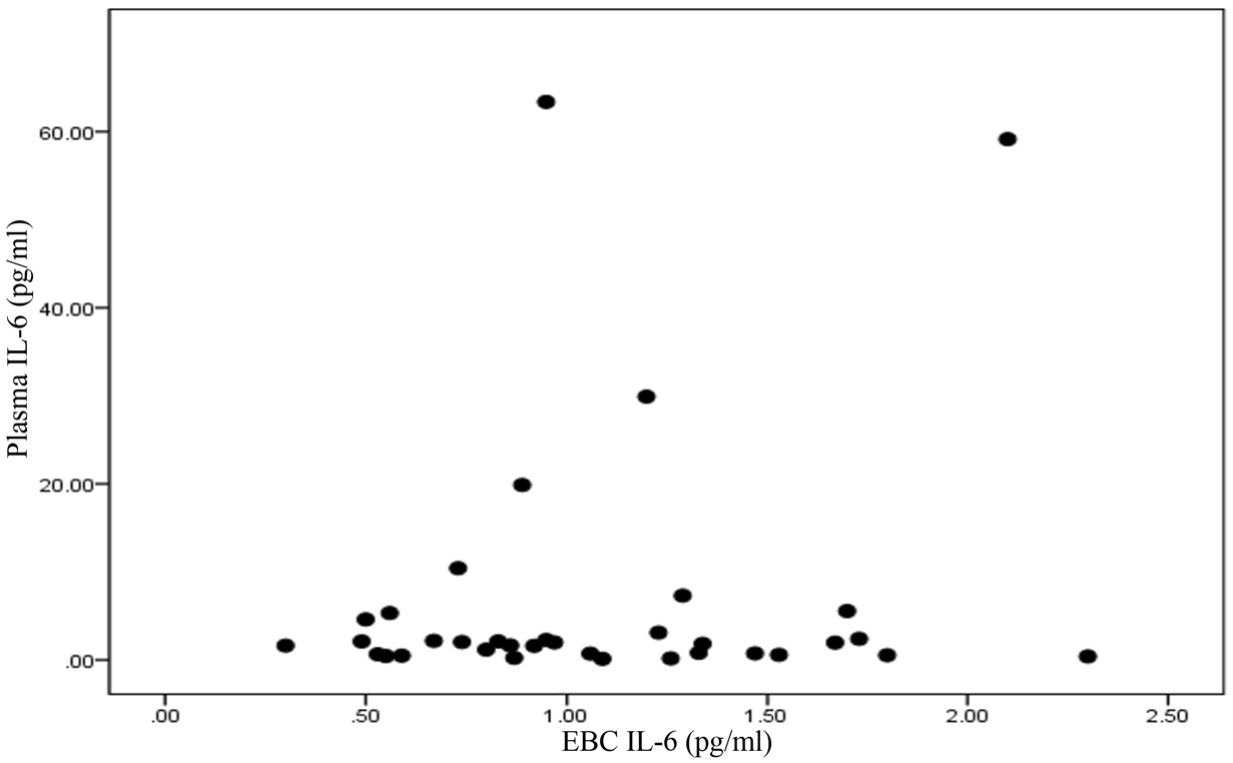
The concentration of IL-6 in Blood and EBC second sampling periods.

The relationship between IL-6 of exhaled breath and IL-6 of plasma were investigated and no significant correlation was found in two periods. In the first period of sampling, the correlation coefficient was calculated to be − 0.046 (p-value = 0.792). In the second period, the correlation coefficient was − 0.024 and p-value was 0.89. No statistically significant correlation was found in this period. There was not observed a significant relationship between IL-6 of exhaled breath and IL-6 of plasma (p-value 0.875 and r 0.028). The concentration of IL-6 in EBC and plasma 1st sampling periods is shown in Fig. 7. Moreover, the concentration of IL-6 in EBC and plasma 2nd sampling periods is shown in Fig. 8.

## Discussion

One of the newest methods for estimating the effects of air pollution in humans is the examination of biological markers in exhaled breath condensate^46,47^. The main advantage of this method is its non-invasiveness and safety even for sensitive individuals, such as children and people with respiratory illness^48–50^. The main limitation of the EBC test is the low concentration of biomarkers compared to other body fluids. Changes in the concentration of markers can be attributed to the quality of condensation, the type of storage, or the sensitivity of the tests, and the data analyses^51–54^. In various studies, it has been reported that EBC collection had no significant difference in the concentration of cytokines at the beginning of the day, mid-day, and mid-week^55^. The results obtained from the school dormitory (Table 2) showed that in ambient air (outdoor), the highest concentration of PM_10_ was recorded in the second sampling period (June) by the value of 66.83 μg/m^3^ and the highest concentrations of PM_2.5_ and PM_1_ were observed in the first period of sampling (March) with a concentration of 24.34 and 16.56 μg/m^3^, respectively.

Observation of high concentrations of PM_10_ during the sampling period may be due to resuspension of dust from the ground and its dispersion due to the wind in the warm season. PM_2.5_ and PM_1_ concentrations in the first sampling period, which occurred in March and the cold season, are higher than those of the second sampling period in the warm season. This difference is due to lack of any inversions in the warm season. The results showed that the highest concentrations of PM_10_ were recorded indoor (in the air of school). Various studies have shown that PM_10_ concentrations in indoor air are heavily influenced by the amount of activity inside. Moreover, when the amount of activity increases indoor, the PM_10_ levels will increase as a result of the resuspension of these particles. It should be noted that in the second period of sampling, the concentration of PM_10_ in ambient air was lower than the air inside, suggesting that the concentration of PM_10_ in indoor air was independent of its concentration in outdoor air and largely depends on the activity of the students living in it.

The average concentration of exposure to PM_2.5_ affects the concentration of these particles in the air. Also, according to the average concentration of PM_1_ exposure in the first sampling period (March) and the second sampling (June) and in comparison with the mean concentration in indoor and outdoor air in the two sampling periods recorded for PM_1_ particles, indoor PM_10_ concentration was recorded significantly higher in the second period of sampling and PM_1_ concentration, both indoor and outdoor, and decreased in the second period of sampling.

The mean concentration of IL-6 in EBC was 0.56 pg/ml in the first period and 1.08 pg/ml in the second period of sampling. The ratio of IL-6 in EBC in the second period of sampling to the first period of sampling in healthy young adults was 1.92. The mean concentration for IL-6 and sTNFRII, in the first sampling period, was recorded at 7.2 and 1730 pg/ml, respectively. The concentration of these markers in the second period of sampling was 13.36 and 1880 pg/ml, respectively. As noted above, no sTNF-RII was detected in EBC. The ratio of IL-6 and sTNFRII in the first period of sampling to the second period of sampling was 1.85 and 1.9, respectively.

In some studies, the level of interleukin-6 and RS-NOs of EBC in patients has been reported more than that of healthy subjects. A significant correlation was found between the coarse particles and plasma sTNF-RII (p-value = 0.001). The results also show that increasing the concentration of PM_10_ increases the WBC. The correlation between coarse particles and WBC was statistically significant (p-value = 0.001). In this study, since WBC and IL-6 of EBC are directly correlated to PM_10_, the increase in coarse particles in the second sampling period may increase the activity of the immune system and eventually increase these parameters in the blood and EBC.

There was no correlation between fine particles and biomarkers. The mean concentration of RS-NOs in the first period of sampling was 0.97 while in the second period was 1.13. Moreover, this ratio was 1.16 for RS-NOs in the second sampling period to the first sampling period. This ratio was 1.16 for RS-NOs in the second sampling period to the first sampling period. There was also no correlation between particulate matter and RS-NOs in EBc. Increasing the concentration of large suspended particles in the second sampling period may increase the activity of the immune system in the body and ultimately increase this parameter in exhaled breath. No small particles are be associated with biomarkers. There was no correlation between the concentrations of particulate matter in exhaled breath with RS-NOs.

In a study conducted by Manney et al., there was a direct correlation between respiratory airborne coarse particles (PM_10_) and biomarkers; however, there was no relationship between other respiratory airborne particles and biomarkers^39^. The results of this study also show that the mean concentration of IL-6 in the blood in the first period of sampling was 12.83 times more than the concentration of IL-6 in EBC. Furthermore, the mean concentration of IL-6 in the blood in the second period of sampling was 12.46 times more than the concentration of IL-6 in EBC. The mean concentration of IL-6 in the two sampling period was 12.66 times more than the concentration of IL-6 in EBC. The results of this study indicate the lack of any significant correlation between EBC IL-6 and plasma IL-6. In a study conducted by Antonopoulou et al., there was no significant correlation between biomarkers in the blood and EBC.

## Conclusion

The present study was designed to investigate the relationship between the concentration of respiratory air particulate matter and inflammatory markers in blood and exhaled breath condensates of students. The results showed that the concentration of PM_10_ in the second period of sampling is more than its concentration in the first period of sampling, and the increase in the concentration of PM_10_ increases the IL-6 in the EBC and also increases the amount of WBC in the blood. WBC and IL-6 of EBC are directly correlated to PM_10_. The increase in coarse particles in the second sampling period may increase the activity of the immune system and eventually increase these parameters in the blood and EBC. There was no correlation between fine particles and biomarkers and no correlation between PM_2.5_ and PM_1_ and biomarkers. The results of this study demonstrate that there is no significant correlation between EBC IL-6 and plasma IL-6. In addition, it was observed that IL-6 concentration in plasma was 12.65 times more than the concentration of IL-6 in EBC. The results showed that by increasing PM_10_ concentration, the concentration of sTNFRII increases in the blood. In this study, no correlation was found between RS-NOs of EBC with particulate matter and no correlation was found between blood IL-6 and particulate matter.
